# Passive Animal Surveillance to Identify Ticks in Wisconsin, 2011–2017

**DOI:** 10.3390/insects10090289

**Published:** 2019-09-08

**Authors:** Xia Lee, Darby S. Murphy, Diep Hoang Johnson, Susan M. Paskewitz

**Affiliations:** 1 Department of Entomology, University of Wisconsin-Madison, Madison, WI 53706, USA; 2 MPH program, University of Wisconsin-Madison, Madison, WI 53706, USA; 3 Infection Control, University of Wisconsin Health, Madison, WI 53715, USA; 4 Department of Entomology, University of Wisconsin-Madison, Madison, WI 53706, USA

**Keywords:** Ixodidae, hard ticks, host record, county record

## Abstract

The introduction of new tick species poses a risk to human and animal health. Systematic active surveillance programs are expensive and uncommon. We evaluated a passive animal surveillance program as a monitoring tool to document the geographic distribution and host associations of ticks in Wisconsin. Passive surveillance partners included veterinary medical clinics, domestic animal shelters, and wildlife rehabilitation centers from 35 of the 72 Wisconsin counties. A total of 10,136 tick specimens were collected from 2325 animals from July 2011 to November 2017 and included *Dermacentor variabilis* Say (29.7% of all ticks), *Ixodes texanus* Banks (25.5%), *Ixodes scapularis* Say (19.5%), *Haemaphysalis leporispalustris* Packard (13.8%), *Ixodes cookei* Packard (4.4%), and *Dermacentor albipictus* Packard (1.7%). Less common species (<1% of collection) included *Ixodes dentatus* Marx, *Ixodes sculptus* Neumann, *Ixodes marxi* Banks, *Amblyomma americanum* Linnaeus, and *Rhipicephalus sanguineus* Latreille. Of the 2325 animals that were examined, most were domestic dogs (53%), eastern cottontail rabbits (16%), domestic cats (15%), and North American raccoons (11%). An additional 21 mammal and 11 bird species were examined at least once during the six years of the study. New county records are summarized for each species. Public health, academic, and veterinary and animal care partners formed a community of practice enabling effective statewide tick surveillance.

## 1. Introduction

Hard ticks (Acari: Ixodidae) are vectors of several pathogens causing diseases that are of medical and veterinary importance. The blacklegged tick, *Ixodes scapularis* Say, is one of the most important hard ticks in the eastern United States as the vector for the etiologic agents causing Lyme disease, anaplasmosis, and babesiosis, among others. Due to its role in vector-borne disease transmission, *I. scapularis* is the only tick species that has been well documented in Wisconsin, using both active and passive surveillance methods [1,2,3,4,5,6,7,8,9,10,11,12,13,14]. These studies demonstrated that *I. scapularis* has undergone a dramatic range expansion in Wisconsin since the 1980s [15]. Another vector species, *Amblyomma americanum* Linnaeus, formerly unknown in Wisconsin, has also been detected with increasing frequency [16,17]. Our current understanding of the presence and geographical range of other ixodid tick species in Wisconsin comes from studies that summarize the results of collections from animals or humans that largely occurred between the early 1900s and the 1970s ([3,6,18,19,20,21,22,23,24,25,26,27,28,29] and references therein). These studies recorded fifteen species, beginning with detection of *Dermacentor albipictus* Packard in 1907. Whether changes have occurred in the diversity and distribution of ticks that do not feed on humans is unknown.

Active tick surveillance, including dragging or flagging, captures important information regarding density and ecological correlates for specific tick species, but such methods are labor intensive and not effective for many species. Passive surveillance, involving the submission of ticks from collaborators or the general public, is a tool that has been successfully implemented to provide information on tick and pathogen distribution as well as host associations in other areas [30,31,32,33,34,35,36,37,38,39,40]. The Wisconsin Department of Health Services and the University of Wisconsin Medical Entomology Laboratory implemented a passive surveillance program in 2011 with the goal of collecting ticks for examination of the extent of the distribution of a newly emerging pathogen, *Ehrlichia muris eauclairensis* [41]. The Surveillance of Wisconsin Animals for Ticks (SWAT) program utilized a network of animal health providers to collect ticks from animals seen at each facility. A variety of animals were sampled, providing an opportunity to update previous records for human- as well as non-human-feeding ticks in Wisconsin.

The objective of this study was to describe the occurrence, distribution, and host associations of ticks resulting from the collection of specimens from domestic and wildlife species by participating veterinary medical clinics, domestic animal shelters, and wildlife rehabilitation centers in Wisconsin.

## 2. Materials and Methods

Veterinarians, domestic animal shelters, and licensed wildlife rehabilitation centers in the state of Wisconsin were contacted by the Wisconsin Department of Health Services by email and telephone for participation in a passive tick surveillance program (SWAT). Collection materials (vials containing ethanol, data sheets, labeled mailers) were provided for all partners. Participants removed ticks from domestic and wild animals and placed all specimens from an individual animal in one 2 mL plastic vial containing 70% ethanol. Participants recorded the name of the facility, the county, the date the tick specimen was removed, the host species on which the tick was found, dog breed (if applicable), exact or approximate location where the tick may have been picked up (where known), travel history (for domestic animals) within the preceding two weeks, approximate number of ticks removed, and any other information the recorder believed important. Samples and datasheets were mailed to the University of Wisconsin–Madison for tick identification.

All tick specimens were identified by use of standard adult, nymphal, and larval taxonomic keys [23,26,42]. Poor condition of some specimens made morphologic identification past the genus level impossible; these specimens were recorded to genus or marked as unknown. Physical specimens are archived at the University of Wisconsin Insect Research Collection. Collection records will be available through the Vector Records Repository at the University of Wisconsin–Madison.

## 3. Results

**Figure 1:**
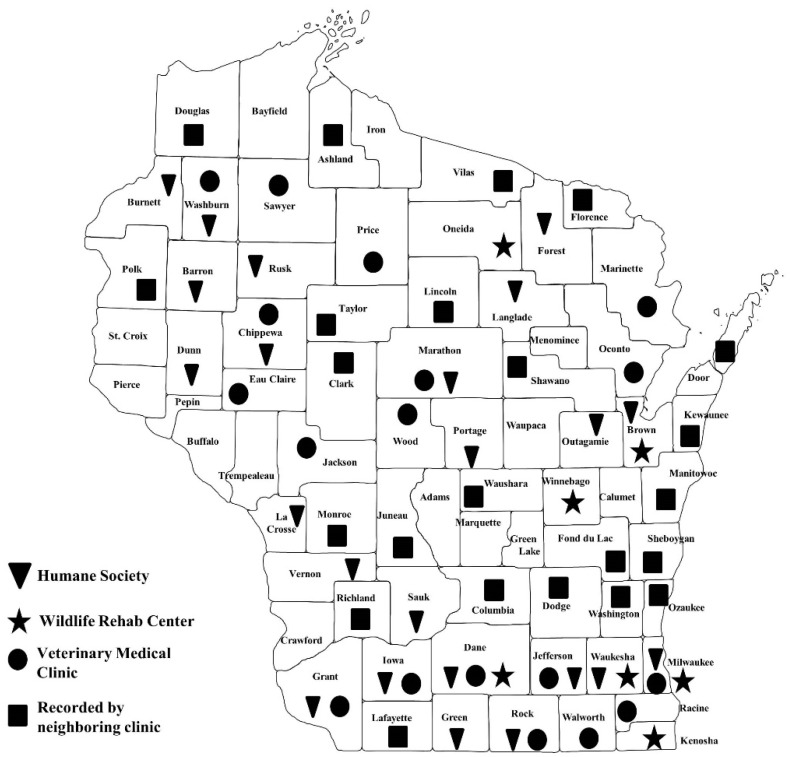
Wisconsin counties where veterinary, humane society, and licensed animal rehabilitators surveyed animals and submitted ticks for identification.

Tick specimens were submitted by 22 veterinary medical clinics, 20 domestic animal shelters, 7 wildlife rehabilitation clinics, and 4 other partners in 35 Wisconsin counties (Figure 1) from August 2011 to November 2017. Most participants were involved for 1–3 years. Partners sometimes collected ticks from animals brought in from neighboring counties and submitted those samples; these counties are identified in Figure 1. Any ticks that were collected from animals with a documented travel history outside of Wisconsin within two weeks of their veterinary visit were excluded from this analysis. A total of 10,136 ticks were removed from 2325 animals of 25 mammal and 11 bird species (Table 1). Of these, the host animal was not recorded for fifty-two samples (255 ticks). Domestic dogs (*Cans familiaris*), domestic cats (*Felis domesticus*), eastern cottontail rabbits (*Sylvilagus floridanus*), and North American raccoons (*Procyon lotor*) were most frequently sampled and yielded 39%, 8%, 30%, and 12% (total of 89%) of all ticks collected, respectively.

The species, stage, sex, and host species of the most commonly collected ticks is summarized in Table 1. Eleven of the sixteen ixodid tick species previously reported from Wisconsin (including *A. americanum*) were collected: *Dermacentor variabilis* Say (3214; 29.7%), *Ixodes texanus* Banks (2588; 25.5%), *I. scapularis* (2463; 19.5%), *Haemaphysalis leporispalustris* Packard (1232; 13.8%), *Ixodes cookei* Packard (461; 4.4%), *D. albipictus* (Packard) (121; 1.7%), *Ixodes dentatus* Marx (27; 0.3%), *Ixodes marxi* Banks (17; 0.2%), *Ixodes sculptus* Neumann (5; 0.05%), *Rhipicephalus sanguineus* Latreille(5; 0.05%) and *A. americanum* (3; 0.03%).

*I. scapularis* had the broadest host range and was collected from 15 mammal and ten bird species, including an adult tick removed from a big brown bat *(Eptesicus fuscus*; new host record) and two larvae removed from a fisher (*Martes pennanti*). *D. variabilis* was collected from 15 mammal species but only one bird, the American Robin (*Turdus migratorius*). *I. cookei* was found on 12 mammal species, including the fisher as well as other members of the canid and mustelid families. *I. texanus* was found on six mammal species, including raccoon, woodchuck (*Marmorata monax*), Virginia opossum (*Didelphis virginiana*), domestic cat, domestic ferret (*Mustela putorius furo*), and an eastern cottontail rabbit; however almost all the specimens were from raccoons (2577 of 2588 collected). Most raccoons had fewer than 50 *I. texanus* but on one occasion, a family of three pups were sampled in Brown County and yield 765 nymphs (June, 2014). *H. leporispalustris* was also found on six species, including two birds, but nearly all of the more than 1000 specimens were from eastern cottontail rabbits. Three tick species were collected from only one or two host species. These included *I. dentatus* (27 adults from Eastern cottontail rabbits), *I. marxi* (seven nymphs, seven adult females, three males; all from raccoons) and *D. albipictus* (121 adults from a farmed elk/white tailed deer operation that did not identify the host species). *I. sculptus* was collected from one raccoon (two nymphs) and three thirteen-lined ground squirrels (*Spermophilus tridecemlineata*; two adult females; one adult male). *A. americanum* was collected from a stray cat and from two dogs, all from southern counties (Jefferson, Dane, Iowa, WI, USA). These associations are consistent with previous host records [23,43,44,45,46,47,48].

**Figure 2:**
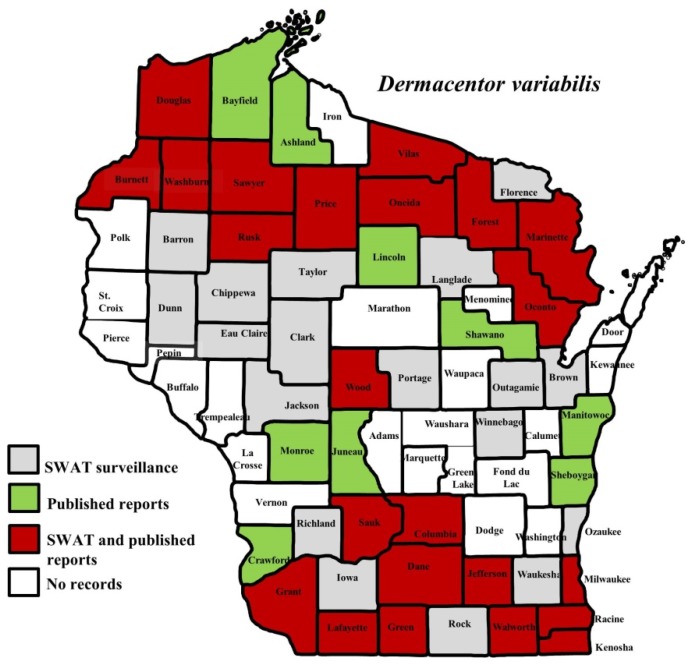
County records for the American dog tick, *Dermacentor variabilis* in Wisconsin [2,3,14,18,27,28].

**Figure 3:**
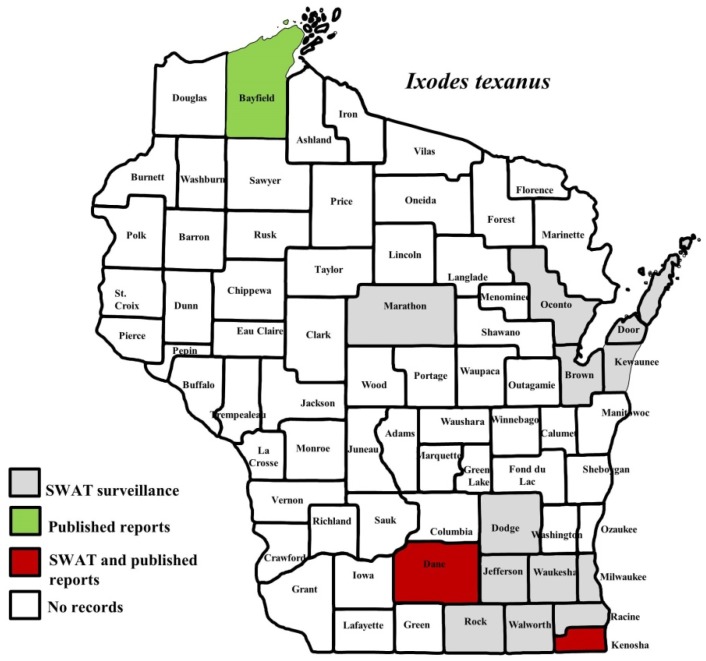
County records for *Ixodes texanus* in Wisconsin [3,27,44].

**Figure 4:**
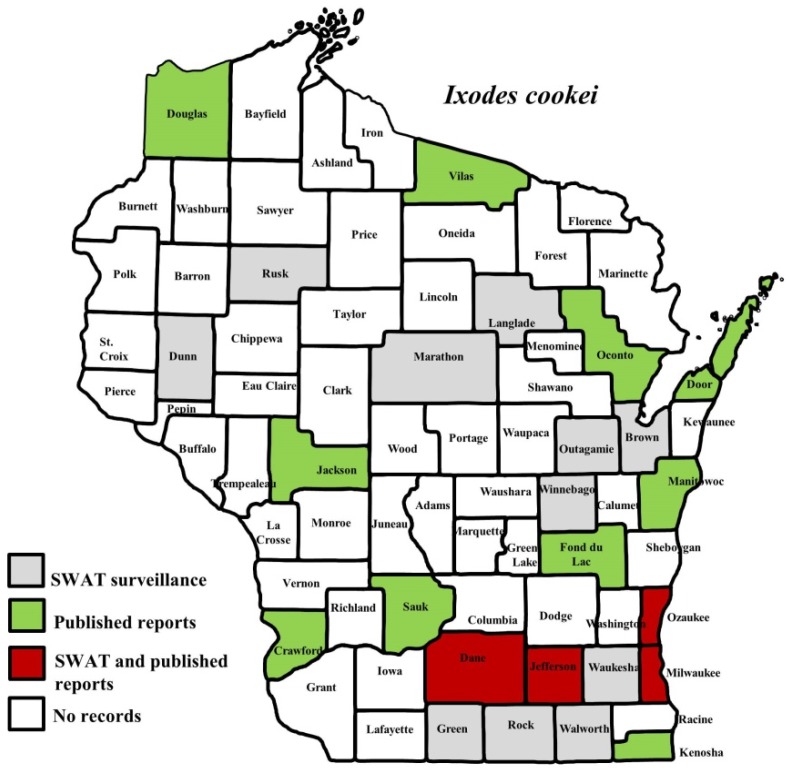
County records for *Ixodes cookei* in Wisconsin [18,27].

**Figure 5:**
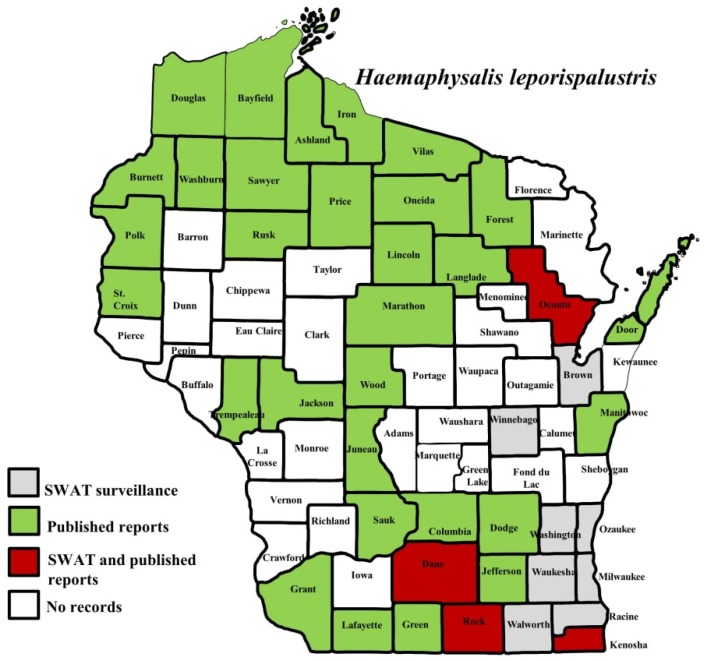
County records for *Haemaphysalis leporispalustris* in Wisconsin [18,27,28].

**Figure 6:**
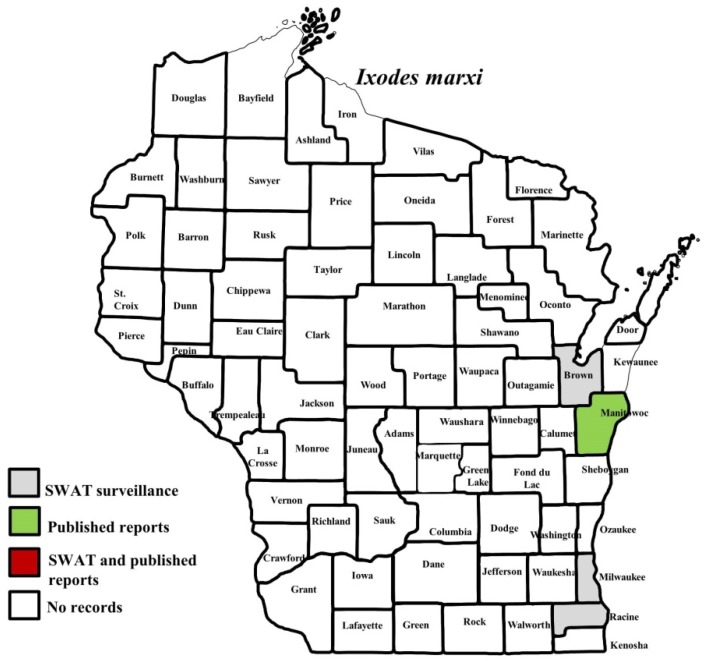
County records for *Ixodes marxi* in Wisconsin [27].

**Figure 7:**
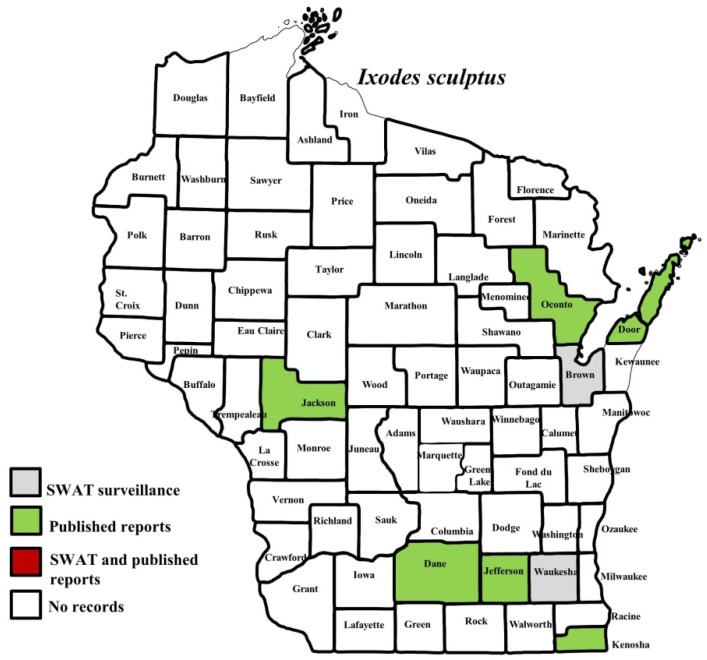
County records for *Ixodes sculptus* in Wisconsin [27].

**Figure 8:**
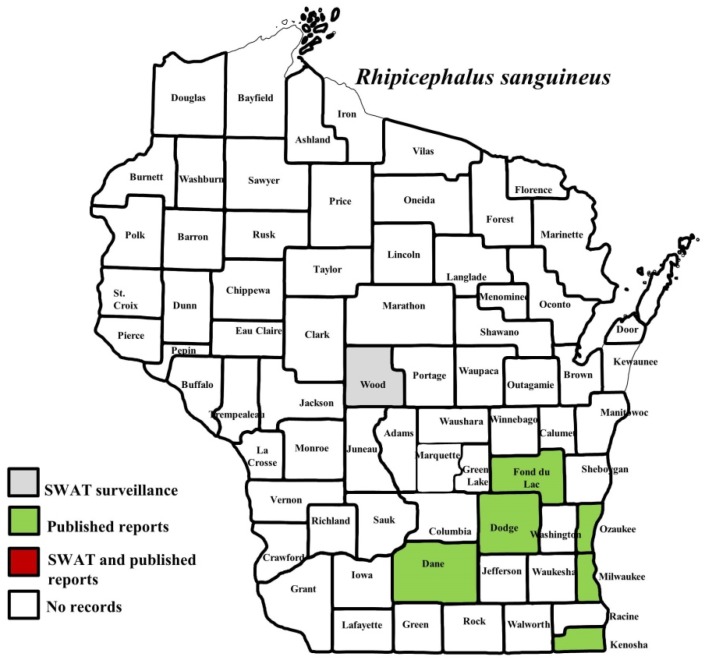
County records for *Rhipicephalus sanguineus* in Wisconsin [3,20,27].

**Figure 9:**
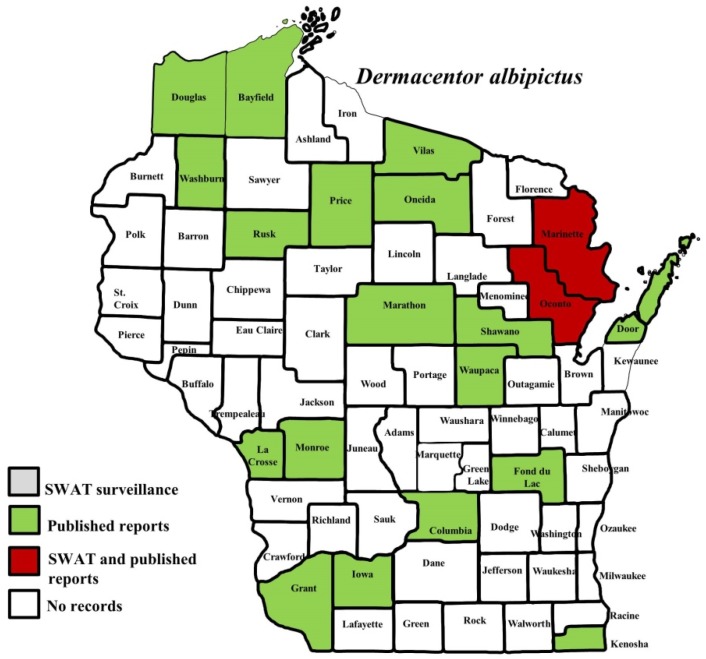
County records for *Dermacentor albipictus* in Wisconsin [1,6,20,27,29].

**Figure 10:**
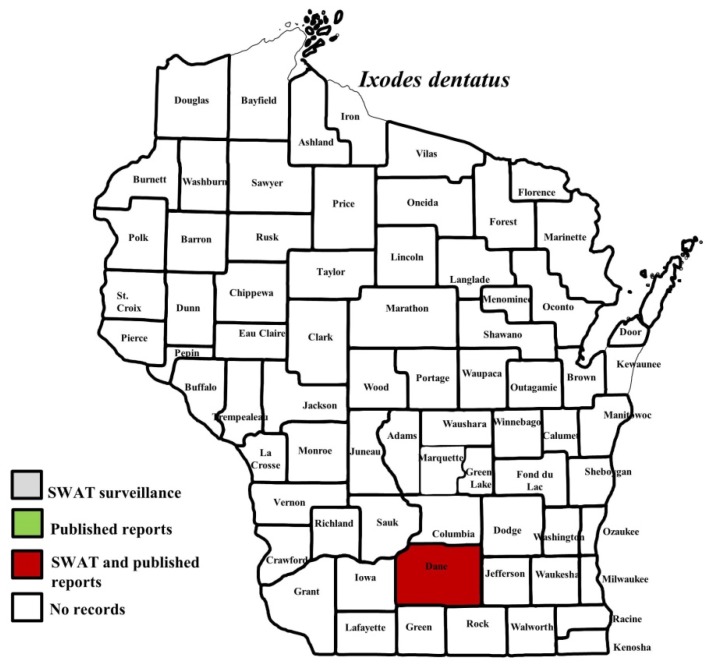
County records for *Ixodes dentatus* in Wisconsin [28].

*I. scapularis* ticks were submitted from 34 of the 35 counties with participating organizations (no specimens submitted from Forest County). Updated distribution maps for this species that included SWAT results were reported in Eisen et al. [15] and we found no additions for the additional 2016–2017 collections included herein. Similarly, *A. americanum* records have been recently summarized [17] and no additions were identified. *D. variabilis* was widely distributed as it was submitted by 32 of the 35 participating counties (no specimens from La Crosse, Vernon, or Marathon Counties) with reports for eight additional nonparticipant counties. We add 18 new county records for this species (Figure 2) to the published record. *I. texanus*, *I. cookei*, and *H. leporispalustris* were submitted from 11, 14, and 10 of the contributing organizations, respectively. We add 12 new county records for *I. texanus* (Figure 3), 11 for *I. cookei* (Figure 4), and 8 for *H. leporispalustris* (Figure 5) to the published record. Fewer collections were made for *I. marxi* (Racine, Milwaukee, Brown Counties, Figure 6), *I. sculptus* (Brown, Waukesha Counties, Figure 7), *R. sanguineus* (Wood County, Figure 8), *D. albipictus* (Marinette and Oconto Counties, Figure 9), and *I. dentatus* (Dane County only, Figure 10). Note that wildlife sampling was most intense in the eastern half of the state where most of the wildlife rehabilitators were located.

## 4. Discussion

Passive tick surveillance is an effective and sensitive method for early detection of the emergence of newly established tick populations and tick-borne disease risk [30,31,40,49], as well as for mapping the geographic distribution and diversity of tick species [36,50]. Significant changes in the distribution and local abundance of multiple tick species have been documented in the last two decades in North America (e.g., *I. scapularis*, *A. americanum*, *Amblyomma maculatum*, *D. variabilis*, *Haemaphysalis longicornis* Neumann) [51] and involvement of veterinary partners provides an effective and engaged network enabling surveys of large geographic areas and greater access to a wide variety of host species.

This study documents the presence of ten tick species collected from 36 different mammal and bird species. The most commonly reported ticks were *I. scapularis* and *D. variabilis* with submissions from 46 of the 72 Wisconsin counties for both species (several partners saw patients from multiple counties). We also recorded a large number of *I. texanus*, *H. leporispalustris*, and *I. cookei*; the majority of these three tick species came from wildlife rehabilitation centers and wildlife sanctuaries located in a more limited set of counties (Brown, Dane, Kenosha, Milwaukee, Oneida, Walworth, Waukesha, and Winnebago Counties). While 97% (992/1020) of all *H. leporispalustris* ticks were collected from eastern cottontail rabbits, we did receive 18 larval tick specimens removed from one cat by the Dane County Humane Society. Our SWAT partners did not note whether this animal was a stray or a domestic cat or whether the animal was in proximity to a rabbit while held at the Humane Society. These ticks were examined by colleagues working with the newly invasive species, *H. longicornis* [52], who were able to confirm the *H. leporispalustris* identification.

This study also documents the submission of two brown dog ticks, *R. sanguineus*, both from dogs. This tick is of interest because of its involvement in the transmission of *Rickettsia rickettsii*, the pathogen causing Rocky Mountain Spotted Fever in the southwestern United States [53]. *R. sanguineus* is not frequently encountered in Wisconsin and we could find few prior published reports of this tick in the state [20,27,54]. Knipping et al. [27] identified three counties with occurrence of this species in the 1940s: Dane, Milwaukee, and Fond du Lac. One of the SWAT submissions was from a facility in a county at the border between Wisconsin and Michigan (Marinette County) in 2011. The tick was on a Rottweiler and was removed in November. The dog owner reported frequent travel between the two states with uncertainty about where the tick was picked up. The other SWAT submission was from Wood County in central Wisconsin in June of 2013 and the owner of the dog did not report travel. In addition to the two SWAT submissions, during 2018 two separate cases of possible introduction of this tick were reported to the Insect Diagnostic Lab in the Department of Entomology at the University of Wisconsin–Madison, with specimens provided for identifications. These were from Barron (northwest Wisconsin) and Dane (southern Wisconsin) Counties. *R. sanguineus* is associated with indoor environments and dog kennels and is more common in the southern and southwestern United States.

The results reported here provide an updated baseline and extend knowledge about the distribution and host associations of the various tick species found in Wisconsin. One important limitation to the study concerns the range of hosts that were surveyed. In Wisconsin, there are 16 recorded ixodid tick species, of which five species were not detected with the SWAT program (*Ixodes muris*, *Ixodes angustus*, *Ixodes banksi*, *Ixodes brunneus*, and *Haemaphysalis chordeilis*). In addition, there were few submissions of four other species (*I. dentatus*, *I. sculptus*, *I. marxi*, and *R. sanguineus*). *I. dentatus* is most commonly found on rabbits; cottontail rabbits were frequently sampled by veterinary partners but *I. dentatus* was only found in Dane County. This confirms a prior report in 1957 where a survey of cottontail rabbits in Wisconsin also found the tick only in Dane county, indicating the northern edge of the range for this species has not changed [28]. With the exception of *R. sanguineus* and *I. dentatus*, the underrepresentation of the other seven ticks is likely a result of the few examinations of their preferred hosts. *I. muris*, *I. angustus*, and *I. marxi* are predominantly found on small rodents such as voles, white-footed mice, deer mice, and shrews [23,55], and both *I. muris* and *I. angustus* have been found through small mammal trapping for studies of Lyme Disease in Wisconsin (unpublished data). *I. sculptus* is often found on small burrowing rodents such as prairie dogs and thirteen-lined ground squirrels; only three thirteen-lined ground squirrels were sampled for the SWAT study [56,57,58]. *I. brunneus* and *H. chordeilis* are ectoparasites of birds [46,59] and relatively few birds were checked by our partners. However, our primary goals were to look for introduced species and to determine host association through passive surveillance by veterinary and wildlife care partners, and as such, we expected a bias towards large and medium sized mammals.

## 5. Conclusions

We provide updated maps of tick species occurrence in Wisconsin based on surveillance of wild and domestic animals. These data provide a valuable baseline enabling evaluation of future changes in tick range and abundance and will be useful in the event of detection of species that are not currently established in Wisconsin.

## Figures and Tables

**Table 1:** Summary of the animals examined for the Surveillance of Wisconsin Animals for Ticks (SWAT) program (2011–2017) and the most commonly collected ixodid ticks.

Host	Common Name for Host	# Of Animals *	*I. scapularis*			*D. variabilis*			*H. leporispalustris*			*I. cookei*			*I. texanus*		
			A	N	L	A	N	L	A	N	L	A	N	L	A	N	L
Mammals																	
*Canis familiaris*	Dog	1204	1619	13	-	2317	4	-	1	-	-	3	1	-	-	-	-
*Canis latrans*	Coyote	6	4	5	-	39	-	-	-	-	-	-	-	-	-	-	-
*Didelphis virginiana*	Opossum	9	-	-	-	112	-	-	-	-	-	-	1	-	1	-	-
*Eptesicus fuscus*	Big brown bat	1	2	-	-	-	-	-	-	-	-	-	-	-	-	-	-
*Equus ferus*	Horse	1	-	-	-	1	-	-	-	-	-	-	-	-	-	-	-
*Erethizon dorsatum*	Porcupine	10	7	-	-	44	-	-	-	-	-	-	-	-	-	-	-
*Felis domesticus*	Cat	350	449	68	3	216	15	4	-	-	18	21	22	6	3	-	-
*Glaucomys volans*	Southern Flying Squirrel	1	-	-	-	-	2	-	-	-	-	-	-	-	-	-	-
*Marmota monax*	Woodchuck	21	7	-	-	4	-	-	-	-	-	54	85	-	3	-	-
*Martes pennanti*	Fisher	1	-	-	2	34	-	-	-	-	-	-	3	-	-	-	-
*Mephitis mephitis*	Striped Skunk	3	-	-	-	4	-	-	-	-	-	6	3	-	-	-	-
*Mustela ermine*	Short-tailed Weasel	1	-	-	-	-	-	-	-	-	-	-	15	-	-	1	1
*Mustela putorius furo*	Ferret	3	-	-	-	3	-	-	-	-	-	2	12	-	-	-	-
*Neovison vison*	Mink	3	-	-	-	-	-	-	-	-	-	29	39	-	-	-	-
*Odocoileus virginianus, Cervus canadensis*	Elk/White-tailed Deer	8	3	2	-	1	1	2	-	-	-	-	-	-	-	-	-
*Ondatra zibethicus*	Muskrat	1	1	-	-	-	-	-	-	-	-	-	-	-	-	-	-
*Peromyscus spp.*	Deer Mice	2	-	-	2	-	-	-	-	-	-	-	-	-	-	-	-
*Procyon lotor*	Raccoon	253	20	55	1	230	-	-	1	-	-	88	34	2	1001	1569	7
*Sciurus carolinensis*	Grey Squirrel	5	1	-	-	10	-	-	-	-	-	1	-	-	-	-	-
*Spermophilus tridecemlineatus*	Thirteen lined Squirrel	3	-	-	-	-	-	-	-	-	-	-	-	-	-	-	-
*Sylvilagus floridanus*	Eastern Cottontail	364	2	4	-	6	5	-	1125	14	63	-	-	-	1	-	-
*Tamias striatus*	Eastern Chipmunk	1	-	3	-	-	-	-	-	-	-	-	-	-	-	-	-
*Tamiasciurus hudsonicus*	Red Squirrel	3	-	10	29	1	-	-	-	-	-	-	-	-	-	-	-
*Urocyon cinereoargenteus*	Gray Fox	1	-	-	-	1	-	-	-	-	-	-	-	-	-	-	-
*Vulpes vulpes*	Red Fox	4	-	-	-	8	-	-	-	-	-	8	8	2	-	-	-
Birds																	
*Bonasa umbellus*	Ruffed Grouse	1	-	17	20	-	-	-	-	-	-	-	-	-	-	-	-
*Buteo jamaicensis*	Red-tailed Hawk	3	1	1	1	-	-	-	-	3	-	-	-	-	-	-	-
*Catharus guttatus*	Hermit Thrush	1	-	1	-	-	-	-	-	-	-	-	-	-	-	-	-
*Corvus corax*	Raven	1	-	3	-	-	-	-	-	-	-	-	-	-	-	-	-
*Grus canadensis*	Sandhill Crane	1	-	4	-	-	-	-	-	-	-	-	-	-	-	-	-
*Meleagris gallopavo*	Wild turkey	1	15	-	-	-	-	-	-	-	-	-	-	-	-	-	-
*Pheucticus ludovicianus*	Rose-breasted Grosbeak	1	-	2	-	-	-	-	-	-	-	-	-	-	-	-	-
*Seiurus aurocapilla*	Ovenbird	1	1	-	-	-	-	-	-	-	-	-	-	-	-	-	-
*Spinus tristis*	American Goldfinch	1	-	1	-	-	-	-	-	-	-	-	-	-	-	-	-
*Turdus migratorius*	American Robin	2	-	1	-	1	-	-	-	-	-	-	-	-	-	-	-
*Zenaida macroura*	Mourning dove	1	-	-	-	-	-	-	1	-	-	-	-	-	-	-	-
*Not identified*	Unknown Animal	52	85	-	-	147	-	-	6	-	-	16	-	-	1	-	-
*Totals*		2325	2217	190	58	3179	27	6	1134	17	81	228	223	10	1010	1570	8

A = Adults, N = Nymphs, L = Larvae; * Number of animals with at least one tick.

## References

[1] French J.B., Schell W.L., Kazmierczak J.J., Davis J.P. (1992). Changes in population density and distribution of *Ixodes dammini* (Acari: Ixodidae) in Wisconsin during the 1980s. J. Med. Entomol..

[2] Jackson J.O., DeFoliart G.R. (1970). *Ixodes scapularis* Say in northern Wisconsin. J. Med. Entomol..

[3] Amin O.M. (1976). Lice, mites, and ticks of southeastern Wisconsin mammals. Great Lakes Entomol..

[4] Davis J.P., Schell W.L., Amundson T.E., Godsey M.S., Spielman A., Burgdorfer W., Barbour A.G., LaVenture M., Kaslow R.A. (1984). Lyme disease in Wisconsin: Epidemiologic, clinical, serologic, and entomologic findings. Yale J. Biol. Med..

[5] Diuk-Wasser M.A., Hoen A.G., Cislo P., Brinkerhoff R., Hamer S.A., Rowland M., Cortinas R., Vourc’h G., Melton F., Hickling G.J. (2012). Human risk of infection with *Borrelia burgdorferi*, the Lyme disease agent, in eastern United States. Am. J. Trop. Med. Hyg..

[6] Riehle M., Paskewitz S.M. (1996). *Ixodes scapularis* (Acari: Ixodidae): Status and changes in prevalence and distribution in Wisconsin between 1981 and 1994 measured by deer surveillance. J. Med. Entomol..

[7] Guerra M., Walker E., Jones C., Paskewitz S., Cortinas M.R., Stancil A., Beck L., Bobo M., Kitron U. (2002). Predicting the risk of Lyme disease: Habitat suitability for *Ixodes scapularis* in the north central United States. Emerg. Infect. Dis..

[8] Michalski M., Rosenfield C., Erickson M., Selle R., Bates K., Essar D., Massung R. (2006). *Anaplasma phagocytophilum* in central and western Wisconsin: A molecular survey. Parasitol. Res..

[9] Paskewitz S.M., Vandermause M., Belongia E.A., Kazmierczak J.J. (2001). *Ixodes scapularis* (Acari: Ixodidae): Abundance and rate of infection with *Borrelia burgdorferi* in four state parks in Wisconsin. J. Med. Entomol..

[10] Coyle D.R., Murphy M.W., Paskewitz S.M., Orrock J.L., Lee X., Murphy R.J., McGeehin M.A., Raffa K.F. (2013). Belowground herbivory in red pine stands initiates a cascade that increases abundance of Lyme disease vectors. For. Ecol. Manag..

[11] Lee X., Hardy K., Johnson D.H., Paskewitz S.M. (2013). Hunter-killed deer surveillance to assess changes in the prevalence and distribution of *Ixodes scapularis* (Acari: Ixodidae) in Wisconsin. J. Med. Entomol..

[12] Caporale D.A., Johnson C.M., Millard B.J. (2005). Presence of *Borrelia burgdorferi* (Spirochaetales: Spirochaetaceae) in southern Kettle Moraine State Forest, Wisconsin, and characterization of strain W97F51. J. Med. Entomol..

[13] Steiner F.E., Pinger R.R., Vann C.N., Grindle N., Civitello D., Clay K., Fuqua C. (2014). Infection and co-infection rates of *Anaplasma phagocytophilum* variants, *Babesia* spp., *Borrelia burgdorferi*, and the rickettsial endosymbiont in *Ixodes scapularis* (Acari: Ixodidae) from sites in Indiana, Maine, Pennsylvania, and Wisconsin. J. Med. Entomol..

[14] French J.B. (1995). *Ixodes scapularis* (Acari: Ixodidae) at the edge of its range in southern Wisconsin. J. Med. Entomol..

[15] Eisen R.J., Eisen L., Beard C.B. (2016). County-scale distribution of *Ixodes scapularis* and *Ixodes pacificus* (Acari: Ixodidae) in the continental United States. J. Med. Entomol..

[16] Springer Y.P., Eisen L., Beati L., James A.M., Eisen R.J. (2014). Spatial distribution of counties in the continental United States with records of occurrence of *Amblyomma americanum* (Ixodida: Ixodidae). J. Med. Entomol..

[17] Christenson M., Lee X., Larson S., Johnson D.H., Jensen J., Meller M., Paskewitz S. (2017). Occurrence of *Amblyomma americanum* (Acari: Ixodidae) and human infection with *Ehrlichia chaffeensis* in Wisconsin, 2008–2015. J. Med. Entomol..

[18] Amin O. (1973). Preliminary survey of vertebrate ectoparasites in southeastern Wisconsin. J. Med. Entomol..

[19] Banks N. (1908). Tick-borne diseases and their origin. J. Econ. Entomol..

[20] Bishopp F.C., Trembley H.L. (1945). Distribution and hosts of certain North American Ticks. J. Parasitol..

[21] Cooley R. (1946). Note on the Tick, Ixodes-Angustus Neumann. J. Parasitol..

[22] Cooley R.A., Kohls G.M. (1945). The genus *Ixodes* in North America. Natl. Inst. Health Bull..

[23] Durden L.A., Keirans J.E. (1996). Nymphs of the Genus Ixodes (Acari: Ixodidae) of the United States: Taxonomy, Identification Key, Distribution, Hosts, and Medical/veterinary Importance.

[24] Godsey M., Amundson T., Burgess E., Schell W., Davis J., Kaslow R., Edelman R. (1987). Lyme disease ecology in Wisconsin—Distribution and host preferences of *Ixodes dammini*, and prevalence of antibody to *Borrelia burgdorferi* in small mammals. Am. J. Trop. Med. Hyg..

[25] Keirans J.E., Clifford C.M. (1978). The genus *Ixodes* in the United States: A scanning electron microscope study and key to the adults. J. Med. Entomol. Suppl..

[26] Keirans J., Litwak T. (1989). Pictorial key to the adults of hard ticks, Family Ixodidae (Ixodida, Ixodoidea), east of the Mississippi River. J. Med. Entomol..

[27] Knipping P.A., Morgan B.B., Dicke R.J. (1950). Notes on the distribution of Wisconsin ticks. Trans. Wis. Acad. Sci. Arts Lett..

[28] Haas G.E. (1957). Ectoparasites of the Mearns Cottontail in Wisconsin. Ph.D. Thesis.

[29] Anderson J., Duray P., Magnarelli L. (1987). Prevalence of *Borrelia burgdorferi* in white-footed mice and *Ixodes dammini* at Fort McCoy, Wisconsin. J. Clin. Microbiol..

[30] Ogden N.H., Trudel L., Artsob H., Barker I.K., Beauchamp G., Charron D.F., Drebot M.A., Galloway T.D., O’Handley R., Thompson R.A. (2006). *Ixodes scapularis* ticks collected by passive surveillance in Canada: Analysis of geographic distribution and infection with Lyme Borreliosis agent *Borrelia burgdorferi*. J. Med. Entomol..

[31] Rand P.W., Lacombe E.H., Dearborn R., Cahill B., Elias S., Lubelczyk C.B., Beckett G.A., Smith R.P. (2007). Passive surveillance in Maine, an area emergent for tick-borne diseases. J. Med. Entomol..

[32] Xu G., Mather T.N., Hollingsworth C.S., Rich S.M. (2016). Passive surveillance of *Ixodes scapularis* (Say), their biting activity, and associated pathogens in Massachusetts. Vector Borne Zoonotic Dis..

[33] Nieto N.C., Porter W.T., Wachara J.C., Lowrey T.J., Martin L., Motyka P.J., Salkeld D.J. (2018). Using citizen science to describe the prevalence and distribution of tick bite and exposure to tick-borne diseases in the United States. PLoS ONE.

[34] Porter W.T., Motyka P.J., Wachara J., Barrand Z.A., Hmood Z., McLaughlin M., Pemberton K., Nieto N.C. (2019). Citizen science informs human-tick exposure in the Northeastern United States. Int. J. Health Geogr..

[35] Tulloch J.S.P., Mcginley L., Sanchez-Vizcaino F., Medlock J.M., Radford A.D. (2017). The passive surveillance of ticks using companion animal electronic health records. Epidemiol. Infect..

[36] Smith R.P., Lacombe E.H., Rand P.W., Dearborn R. (1992). Diversity of tick species biting humans in an emerging area for Lyme disease. Am. J. Public Health.

[37] Schillberg E., Lunny D., Lindsay L.R., Nelder M.P., Russell C., Mackie M., Coats D., Berry A., Hoon K.N.Y. (2018). Distribution of *Ixodes scapularis* in northwestern Ontario: Results from active and passive surveillance activities in the northwestern health unit catchment area. Int. J. Environ. Res. Public Health.

[38] Ripoche M., Gasmi S., Adam-Poupart A., Koffi J.K., Lindsay L.R., Ludwig A., Milord F., Ogden N.H., Thivierge K., Leighton P.A. (2018). Passive tick surveillance provides an accurate early signal of emerging Lyme disease risk and human cases in southern Canada. J. Med. Entomol..

[39] Gasmi S., Ogden N.H., Ripoche M., Leighton P.A., Lindsay R.L., Nelder M.P., Rees E., Bouchard C., Vrbova L., Rusk R. (2019). Detection of municipalities at-risk of Lyme disease using passive surveillance of *Ixodes scapularis* as an early signal: A province-specific indicator in Canada. PLoS ONE.

[40] Koffi J.K., Leighton P.A., Pelcat Y., Trudel L., Lindsay L.R., Milord F., Ogden N.H. (2012). Passive surveillance for *Ixodes scapularis* ticks: Enhanced analysis for early detection of emerging Lyme disease risk. J. Med. Entomol..

[41] Murphy D.S., Lee X., Larson S.R., Johnson D.K.H., Loo T., Paskewitz S.M. (2017). Prevalence and distribution of human and tick infections with the *Ehrlichia muris*-like agent and *Anaplasma phagocytophilum* in Wisconsin, 2009–2015. Vector Borne Zoonotic Dis..

[42] Clifford C.M., Anastos G., Elbl A. (1961). The Larval Ixodid Ticks of the Eastern United States (Acarina-Ixodidae).

[43] Cohen S.B., Freye J.D., Dunlap B.G., Dunn J.R., Jones T.F., Moncayo A.C. (2010). Host associations of *Dermacentor*, *Amblyomma*, and *Ixodes* (Acari: Ixodidae) ticks in Tennessee. J. Med. Entomol..

[44] Darsie R.F., Anastos G. (1957). Geographical distribution and hosts of *Ixodes texanus* Banks (Acarina, Ixodidae). Ann. Entomol. Soc. Am..

[45] Hertz J.C., Clemons B.C.F., Lord C.C., Allan S.A., Kaufman P.E. (2017). Distribution and host associations of ixodid ticks collected from wildlife in Florida, USA. Exp. Appl. Acarol..

[46] Kollars T.M., Oliver J.H. (2003). Host associations and seasonal occurrence of *Haemaphysalis leporispalustris*, *Ixodes brunneus*, *I. cookei*, *I. dentatus*, and *I. texanus* (Acari: Ixodidae) in Southeastern Missouri. J. Med. Entomol..

[47] Keirans J.E., Hutcheson H.J., Durden L.A., Klompen J.S.H. (1996). *Ixodes* (*Ixodes*) *scapularis* (Acari: Ixodidae): Redescription of all active stages, distribution, hosts, geographical variation, and medical and veterinary importance. J. Med. Entomol..

[48] Lubelczyk C., Lacombe E.H., Elias S.P., Beati L., Rand P.W., Smith R.P. (2014). Parasitism of mustelids by ixodid ticks (Acari: Ixodidae), Maine and New Hampshire, USA. Ticks Tick Borne Dis..

[49] Ogden N.H., Bouchard C., Kurtenbach K., Margos G., Lindsay L.R., Trudel L., Nguon S., Milord F. (2010). Active and passive surveillance and phylogenetic analysis of *Borrelia burgdorferi* elucidate the process of Lyme disease risk emergence in Canada. Environ. Health Perspect..

[50] Walker E.D., Stobierski M.G., Poplar M.L., Smith T.W., Murphy A.J., Smith P.C., Schmitt S.M., Cooley T.M., Kramer C.M. (1998). Geographic distribution of ticks (Acari: Ixodidae) in Michigan, with emphasis on *Ixodes scapularis* and *Borrelia burgdorferi*. J. Med. Entomol..

[51] Sonenshine D.E. (2018). Range expansion of tick disease vectors in North America: Implications for spread of tick-borne disease. Int. J. Environ. Res. Public Health.

[52] Rainey T., Occi J.L., Robbins R.G., Egizi A. (2018). Discovery of *Haemaphysalis longicornis* (Ixodida: Ixodidae) parasitizing a sheep in New Jersey, United States. J. Med. Entomol..

[53] Demma L.J., Traeger M.S., Nicholson W.L., Paddock C.D., Blau D.M., Eremeeva M.E., Dasch G.A., Levin M.L., Singleton J.J., Zaki S.R. (2005). Rocky mountain spotted fever from an unexpected tick vector in Arizona. N. Engl. J. Med..

[54] Cooley R.A. (1946). The Genera Boophilus, Rhipicephalus, and Haemaphysalis (Ixodidae) of the New World.

[55] Murrell B.P., Durden L.A., Cook J.A. (2003). Host associations of the tick, *Ixodes angustus* (Acari: Ixodidae), on Alaskan mammals. J. Med. Entomol..

[56] Miller R.S., Ward R.A. (1960). Ectoparasites of pocket gophers from Colorado. Am. Midl. Nat..

[57] Brillhart D., Fox L., Upton S. (1994). Ticks (Acari, Ixodidae) collected from small and medium-sized Kansas mammals. J. Med. Entomol..

[58] Salkeld D.J., Eisen R.J., Antolin M.F., Stapp P., Eisen L. (2006). Host usage and seasonal activity patterns of *Ixodes kingi* and *I. sculptus* (Acari: Ixodidae) nymphs in a Colorado prairie landscape, with a summary of published North American host records for all life stages. J. Vector Ecol..

[59] Kohls G. (1960). Records and new synonymy of New World *Haemaphysalis* ticks, with descriptions of the nymph and larva of *H. juxtakochi* Cooley. J. Parasitol..

